# Behavioral data of thin-film single junction amorphous silicon (a-Si) photovoltaic modules under outdoor long term exposure

**DOI:** 10.1016/j.dib.2016.02.055

**Published:** 2016-02-27

**Authors:** Sofiane Kichou, Santiago Silvestre, Gustavo Nofuentes, Miguel Torres-Ramírez, Aissa Chouder, Daniel Guasch

**Affiliations:** a MNT Group, Electronic Engineering Department, Universitat Politécnica de Catalunya (UPC) BarcelonaTech, C/Jordi Girona 1-3, Campus Nord UPC, 08034 Barcelona, Spain; b IDEA Research Group, University of Jaén, Campus de Las Lagunillas, 23071 Jaén, Spain; c Univ. M׳sila, Fac. Technologies, Dep. Génie Electrique, BP 166 Ichbelia, 28000 M׳sila, Algeria; d Departament d׳EnginyeriaTelemàtica, Universitat Politécnica de Catalunya (UPC) BarcelonaTech. EDIFICI VG1 (EPSEVG), Avda. Víctor Balaguer, 1, 08800 Vilanova i la Geltrú, Spain

**Keywords:** Light-induced degradation (LID), a-Si PV modules, Stabilization period

## Abstract

Four years׳ behavioral data of thin-film single junction amorphous silicon (a-Si) photovoltaic (PV) modules installed in a relatively dry and sunny inland site with a Continental-Mediterranean climate (in the city of Jaén, Spain) are presented in this article. The shared data contributes to clarify how the Light Induced Degradation (LID) impacts the output power generated by the PV array, especially in the first days of exposure under outdoor conditions. Furthermore, a valuable methodology is provided in this data article permitting the assessment of the degradation rate and the stabilization period of the PV modules.

Further discussions and interpretations concerning the data shared in this article can be found in the research paper “Characterization of degradation and evaluation of model parameters of amorphous silicon photovoltaic modules under outdoor long term exposure” (Kichou et al., 2016) [1].

**Specifications Table**

**Table:** 

Subject area	*Renewable energy*

More specific subject area	*Photovoltaic systems, a-Si PV module degradation analysis*
Type of data	*Excel table, Matlab figures*
How data was acquired	*– DC voltage and current are recorded at the SMA™ Sunny Boy SB1200 inverter input.*
*– The in-plane irradiance comes from a Kipp & Zonen™ CMP21 pyranometer.*
*– Two Pt 100 resistive thermal detectors (RTD) are used as module temperature sensors being glued to the rear surface of the PV modules.*
Data format	*Analyzed*
Experimental factors	*N/A*
Experimental features	*Data were taken at 5-min intervals from onsite measurements*
Data source location	*Jaén university (Spain), Latitude: 37° 47′14.35′′N*, *Longitude: 3° 46′ 39.73′′W, Altitude: 511 m*
Data accessibility	*Data is within this article*

**Value of the data**

• The shared data may be useful to understand the behavior of thin film single junction amorphous silicon (a-Si) photovoltaic modules based technology deployed under outdoor conditions.
• Understanding the behavior and the characteristics of the a-Si PV modules are essential to improve the reliability of this PV modules based technology and selecting the best technology for the appropriate climatic conditions.
• The methodology used in this data article is valuable for determining the performance and estimating the degradation period of PV modules.

## 1 Data

The shared data describes the behavior of the amorphous silicon (a-Si) thin film PV modules deployed under outdoor conditions from July 2011 to December 2014.

**Fig. 1:**
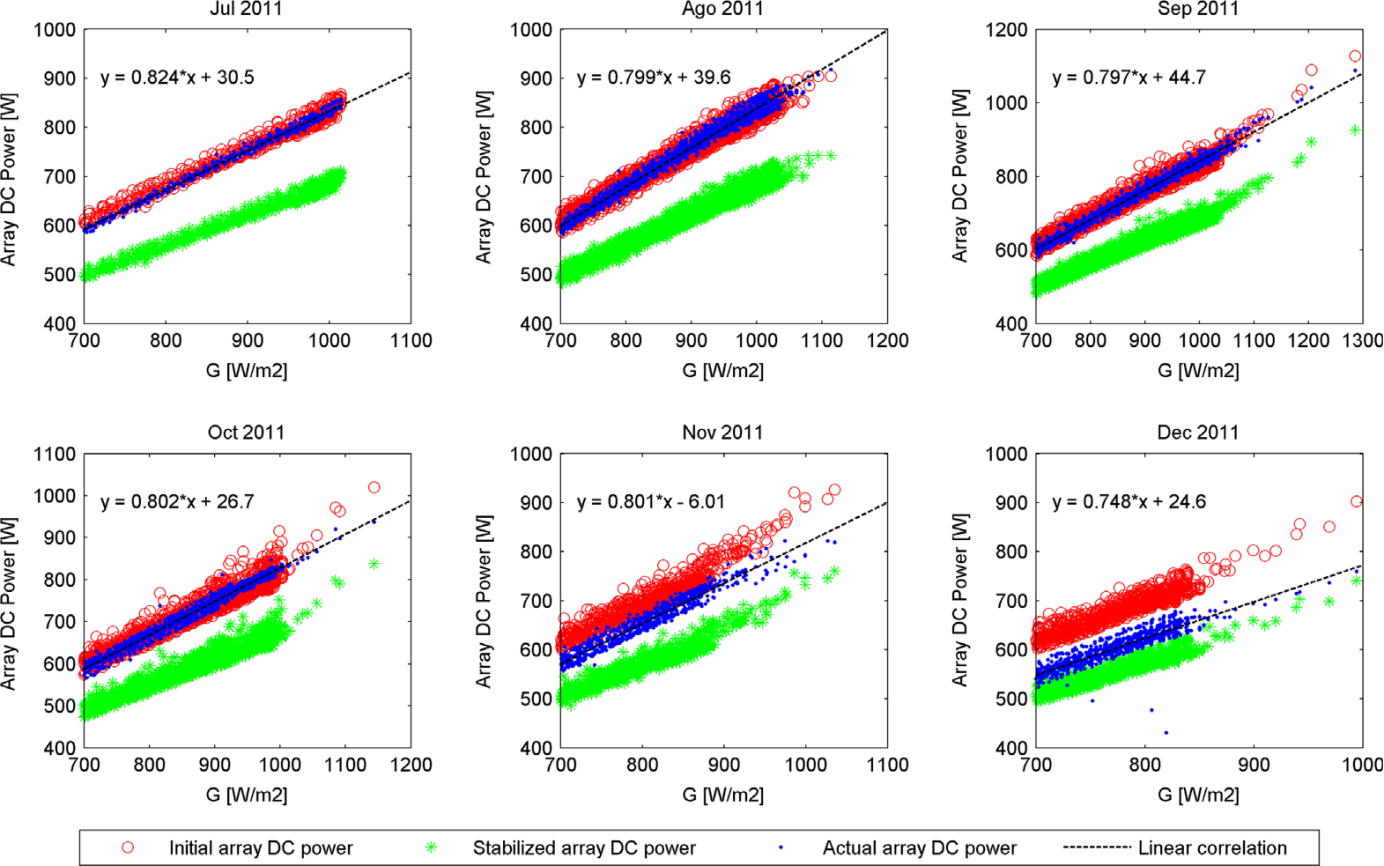
Array DC output powers evolution from July 2011 to December 2011.

**Fig. 2:**
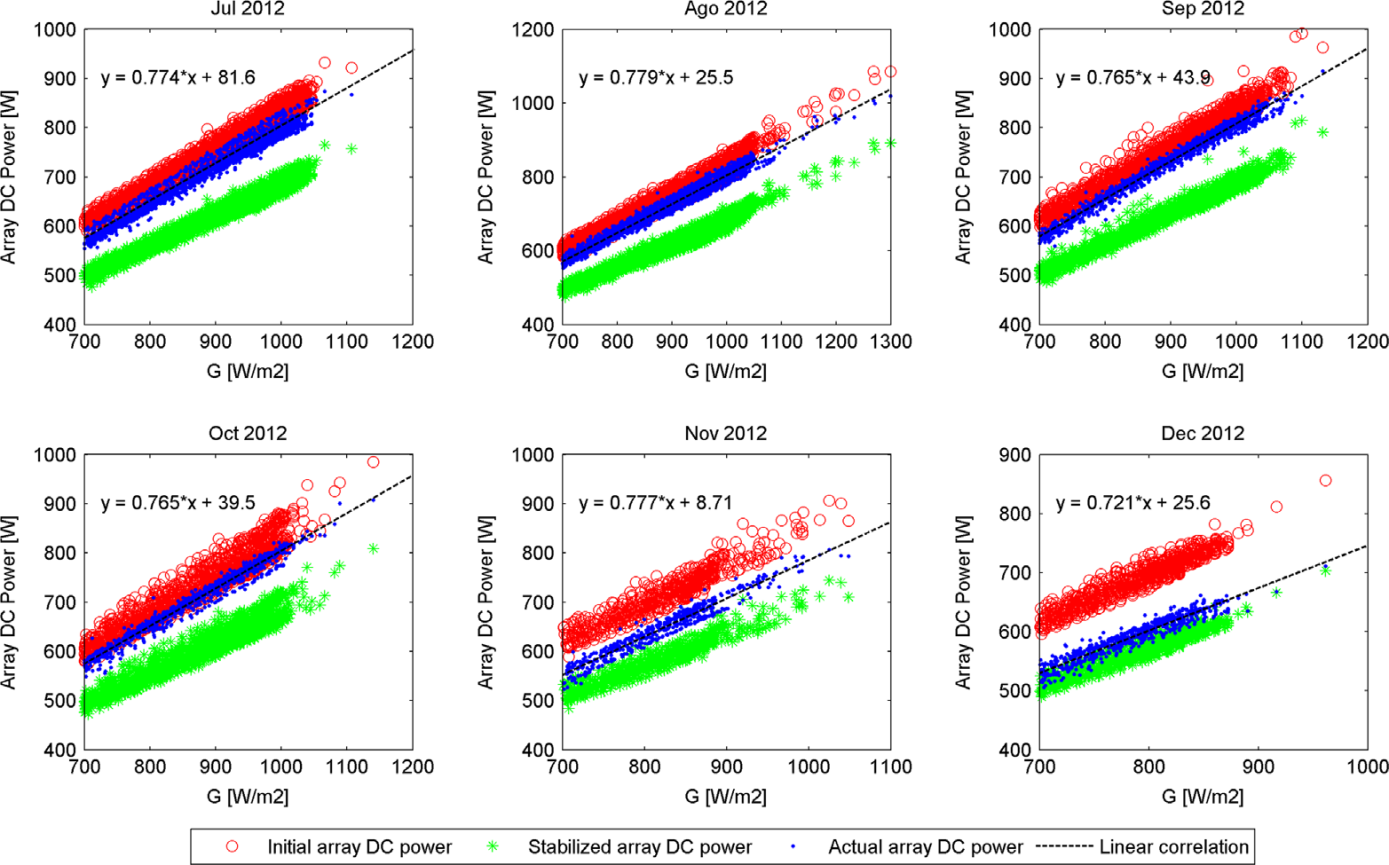
Array DC output powers evolution from July 2012 to December 2012.

**Fig. 3:**
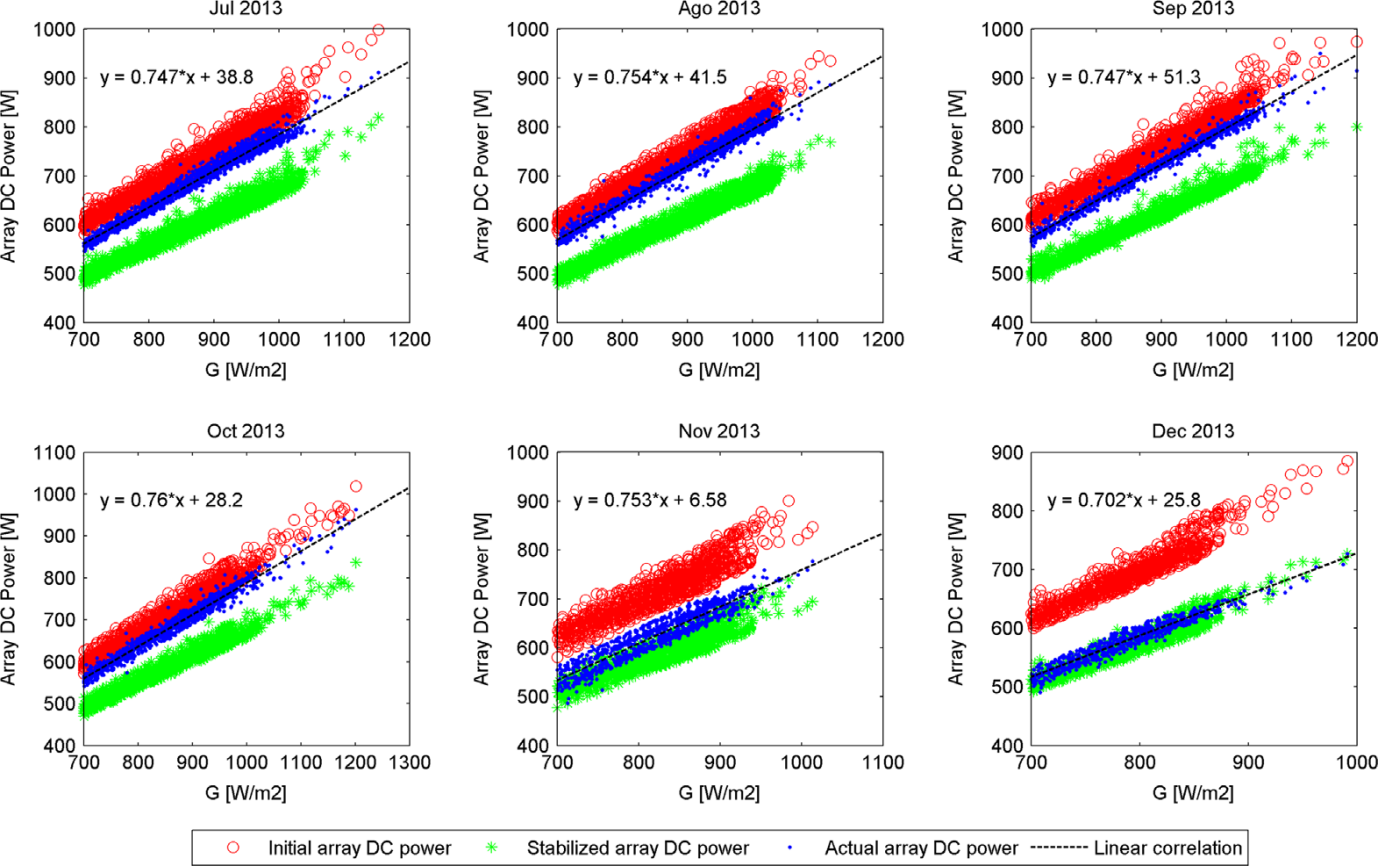
Array DC output powers evolution from July 2013 to December 2013.

**Fig. 4:**
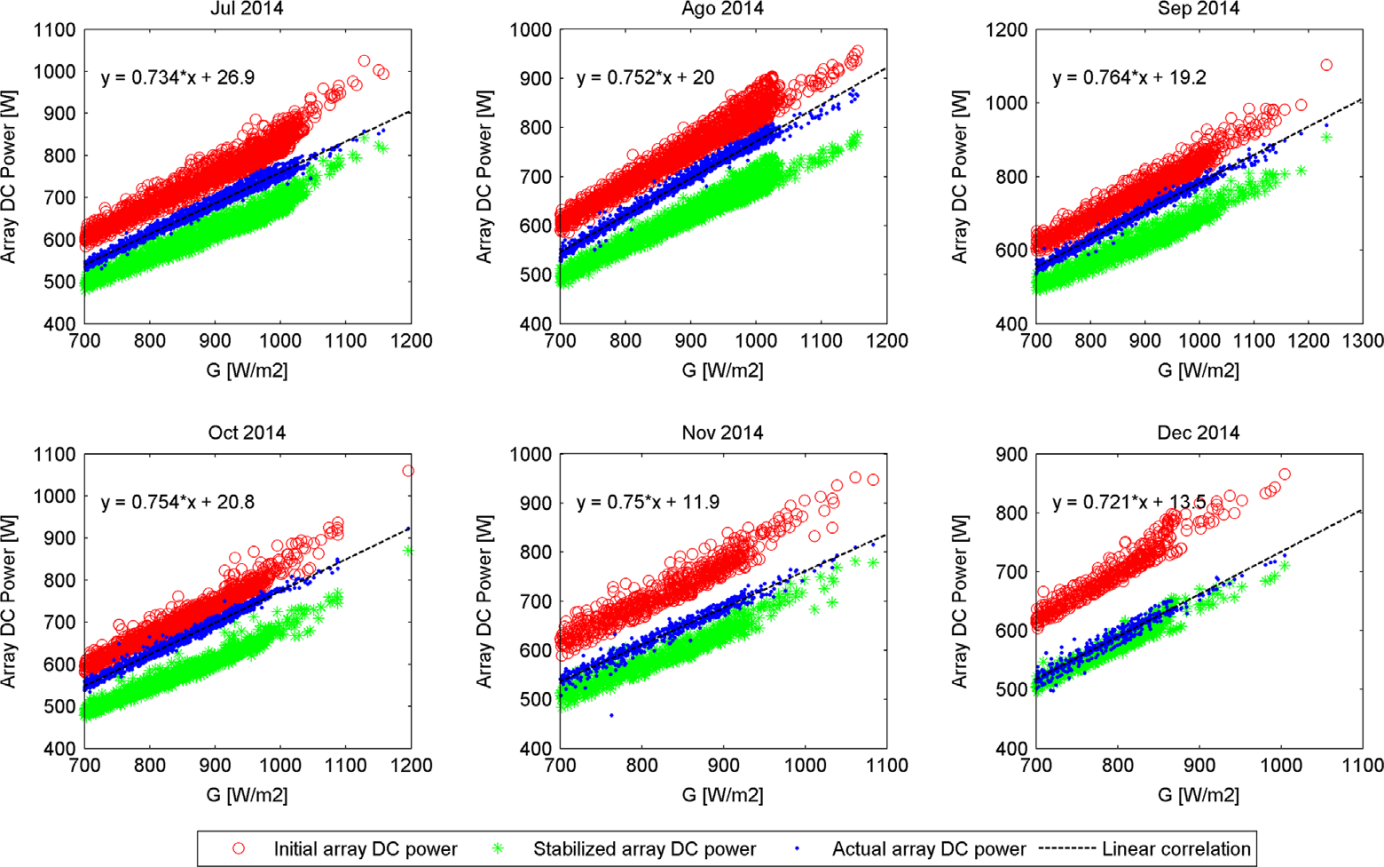
Array DC output powers evolution from July 2014 to December 2014.

The actual DC output power generated by the PV array versus filtered values of irradiance (*G*≥700 W/m²), delimited by two boundaries defined as initial and stable PV array DC output powers for the second semester of each year along the experimental campaign are presented in Fig. 1, Fig. 2, Fig. 3, Fig. 4. The rest of the data figures can be found in the Supplementary material included in this article.

Table 1 illustrates the empirical equations obtained by the Linear Correlation Approach (LCA) applied to the real PV array DC output power permitting the identification of the degradation and stabilization period.

## 2 Experimental design, materials and methods

### 2.1 Photovoltaic system description

The PV system providing the experimental data support presented within this article, is installed in Jaén situated in a dry and sunny inland site, with a Continental-Mediterranean climate. A detailed description of the PV system as well as the climate characterization can be found in [1].

### 2.2 Methodology

The a-Si PV modules present light-induced degradation (LID) due to the Staebler–Wronski effect (SWE) [2], [3], [4]. Several works have been conducted in attempt to explain the real performance characterization of the a-Si PV modules when deployed outdoors.

The degradation rate assessment can be based on the comparison of the monitoring outdoor performance with initial indoor measurements taken as references [5], [6], or by applying Linear Regression (LR) and Classical Seasonal Decomposition (CSD) methods with temperature correction [7], [8].

The data presented in this article were analyzed using the technique proposed by Hussin et al. [9], this method permit assessing the degradation of PV modules exposed under outdoors conditions in terms of power line transition in between two boundaries indicators; Predicted initial and stabilized data values of PV array DC output powers.

To avoid problems of uncertainties caused by low values of irradiance due to the presence of shading, a data filtering process is needed, as explained in [1].

The predicted initial and stabilized data values depend on the measured plane-of-array irradiance (*G*), module temperature (*T*_*c*_), and can be calculated by using the following equations:

(1) $${\mathrm{Pdc}}_{\mathrm{init}}=N_{s}{.N}_{p}.{\mathrm{Pm}}_{\mathrm{init}}.\eta .G_{\mathrm{eff}}.\left(1+\mathrm{kv}.\Delta T\right).\left(1-\mathrm{ki}.\Delta T\right)$$

(2) $${\mathrm{Pdc}}_{\mathrm{stab}}=N_{s}{.N}_{p}.{\mathrm{Pm}}_{\mathrm{stab}}.\eta .G_{\mathrm{eff}}.\left(1+\mathrm{kv}.\Delta T\right).\left(1-\mathrm{ki}.\Delta T\right)$$

(3) $$G_{\mathrm{eff}}=\frac{G}{G_{n}}$$

(4) $$\Delta T=T_{c}-T_{n}$$

where *Pdc*_*init*_ is the predicted array DC power referred to initial (W), *N*_*s*_ and *N*_*p*_ are the number of modules connected in series and parallel respectively, *Pm*_*init*_ is the initial measured peak power of PV module (Wp), *kv* and *ki* are the voltage and current temperature coefficients respectively provided in the manufacturer’s data sheet (1/°C), *Pdc*_*stab*_ is the predicted array DC power referred to stabilized (W), *Pm*_*stab*_ is the stabilized peak power of the PV module found in the manufacturer׳s data sheet (Wp), *η* is the efficiency referred to all general system losses which changes between 0.89 in summer and 0.86 in winter months, *G*_*n*_ and *T*_*n*_ are the reference irradiance and cell temperature respectively under STC (*G*_*n*_=1000 W/m^2^, *T*_*n*_=25 °C).

The empirical equations presented in Table 1 were obtained using a Linear Correlation Approach (LCA) to the actual PV array DC power output in parallel with plotting power versus irradiance [9]. Using the measured gradient LCA, the monthly gradient values can be plotted to observe the degradation and determine the stabilization period upon this type of a-Si PV module technology.

## Figures and Tables

**Table 1:** Monthly empirical equations based Linear Correlation Approach (LCA).

**Sample no.**	**Month**	**Empirical equation**	***R***^**2**^	**Gradient**
**1**	Jul-11	*Pdc*=0.824 *G*+30.5	0.993	0.824
**2**	Aug-11	*Pdc*=0.799 *G*+39.6	0.982	0.799
**3**	Sep-11	*Pdc*=0.797 *G*+44.7	0.987	0.797
**4**	Oct-11	*Pdc*=0.802 *G*+26.7	0.982	0.802
**5**	Nov-11	*Pdc*=0.801 *G*−6.01	0.941	0.801
**6**	Dec-11	*Pdc*=0.74*8 G*+24	0.878	0.748
**7**	Jan-12	*Pdc*=0.755 *G*+1.67	0.941	0.755
**8**	Feb-12	*Pdc*=0.712 *G*+16.4	0.963	0.712
**9**	Mar-12	*Pdc*=0.727 *G*+19.5	0.983	0.727
**10**	Apr-12	*Pdc*=0.727 *G*+26	0.987	0.727
**11**	May-12	*Pdc*=0.723 *G*+47.7	0.966	0.723
**12**	Jun-12	*Pdc*=0.762 *G*+30.6	0.974	0.762
**13**	Jul-12	*Pdc*=0.774 *G*+81.6	0.968	0.774
**14**	Aug-12	*Pdc*=0.779 *G*+25.5	0.973	0.779
**15**	Sep-12	*Pdc*=0.785 *G*+43.9	0.985	0.785
**16**	Oct-12	*Pdc*=0.765 *G*+39.5	0.978	0.765
**17**	Nov-12	*Pdc*=0.770 *G*+8.71	0.941	0.770
**18**	Dec-12	*Pdc*=0.721 *G*+25.6	0.916	0.721
**19**	Jan-13	*Pdc*=0.708 *G*+22.5	0.958	0.708
**20**	Feb-13	*Pdc*=0.699 *G*+22.4	0.978	0.699
**21**	Mar-13	*Pdc*=0.696 *G*+21.5	0.979	0.696
**22**	Apr-13	*Pdc*=0.689 *G*+41.5	0.965	0.689
**23**	May-13	*Pdc*=0.717 *G*+31.1	0.984	0.717
**24**	Jun-13	*Pdc*=0.711 *G*+44.8	0.953	0.711
**25**	Jul-13	*Pdc*=0.747 *G*+38.8	0.977	0.747
**26**	Aug-13	*Pdc*=0.754 *G*+41.5	0.980	0.754
**27**	Sep-13	*Pdc*=0.747 *G*+51.3	0.987	0.747
**28**	Oct-13	*Pdc*=0.760 *G*+28.2	0.973	0.760
**29**	Nov-13	*Pdc*=0.753 *G*+65.8	0.877	0.753
**30**	Dec-13	*Pdc*=0.702 *G*+25.8	0.932	0.702
**31**	Jan-14	*Pdc*=0.677 *G*+39.9	0.972	0.677
**32**	Feb-14	*Pdc*=0.705 *G*+9.83	0.968	0.675
**33**	Mar-14	*Pdc*=0.685 *G*+21.7	0.982	0.685
**34**	Apr-14	*Pdc*=0.704 *G*+25.4	0.983	0.704
**35**	May-14	*Pdc*=0.718 *G*+26.1	0.979	0.718
**36**	Jun-14	*Pdc*=0.720 *G*+31.3	0.980	0.720
**37**	Jul-14	*Pdc*=0.734 *G*+26.9	0.978	0.734
**38**	Aug-14	*Pdc*=0.752 *G*+20	0.986	0.752
**39**	Sep-14	*Pdc*=0.764 *G*+19.2	0.986	0.764
**40**	Oct-14	*Pdc*=0.754 *G*+20.8	0.981	0.754
**41**	Nov-14	*Pdc*=0.750 *G*+11.9	0.964	0.750
**42**	Dec-14	*Pdc*=0.721 *G*+13.5	0.945	0.721
